# Endoscopic submucosal dissection for early gastric cancer in a patient after left ventricular assist device implantation: A case report

**DOI:** 10.1002/deo2.316

**Published:** 2023-11-14

**Authors:** Shinji Kuriki, Yoshiki Tsujii, Hirotsugu Saiki, Takahiro Amano, Ryotaro Uema, Minoru Kato, Takeo Yoshihara, Yoshito Hayashi, Hayato Hikita, Tetsuo Takehara

**Affiliations:** ^1^ Department of Gastroenterology and Hepatology Osaka University Graduate School of Medicine Osaka Japan

**Keywords:** antithrombotic drug, cerebral infarction, early gastric cancer, endoscopic submucosal dissection, ventricular assist device

## Abstract

The use of left ventricular assist device (LVAD) implantation has increased in recent years. Here, we report the first case of gastric endoscopic submucosal dissection (ESD) following LVAD implantation. A 69‐year‐old man who previously underwent LVAD implantation for severe heart failure underwent esophagogastroduodenoscopy, which revealed a 15‐mm flat‐elevated cancerous lesion at the greater curvature of the gastric angle. Before ESD, antithrombotic drugs were discontinued and replaced with 10,000 units of heparin. However, on the second day, the patient experienced dysarthria and right upper‐extremity movement disorder despite a prothrombin time/international normalized ratio (PT‐INR) of 2.01. On the fifth day, computed tomography revealed a low‐density area extending from the left corona radiata to the basal ganglia, leading to a diagnosis of acute cerebral infarction. Aspirin and warfarin were immediately restarted, while the heparin infusion was discontinued after confirming recovery of PT activity. Thereafter, the neurological abnormalities did not aggravate and a trend toward symptomatic improvement was observed. Two months later, ESD was performed under continuous warfarin administration (PT‐INR, 2.62) without heparin replacement, and the lesion was curatively resected without complications. The patient was discharged without adverse events. This case report provides useful information on the feasibility and perioperative management of ESD in patients with LVAD.

## INTRODUCTION

Currently, left ventricular assist device (LVAD) implantation is not only used for bridge‐to‐transplantation purposes but also as destination therapy (DT) because it improves the prognosis and quality of life of patients with severe heart failure.^1^ Some patients who undergo LVAD implantation as bridge‐to‐transplantation may eventually use it as DT.^1^ Therefore, the duration of LVAD implantation is expected to increase in the future, as is the frequency of primary malignancies of the gastrointestinal tract in patients with LVAD. Although the safety of non‐cardiac surgery for patients with LVAD has been reported, endoscopic submucosal dissection (ESD) remains unreported. To our knowledge, this is the first report of gastric ESD in a patient after LVAD implantation.

## CASE REPORT

**FIGURE 1:**
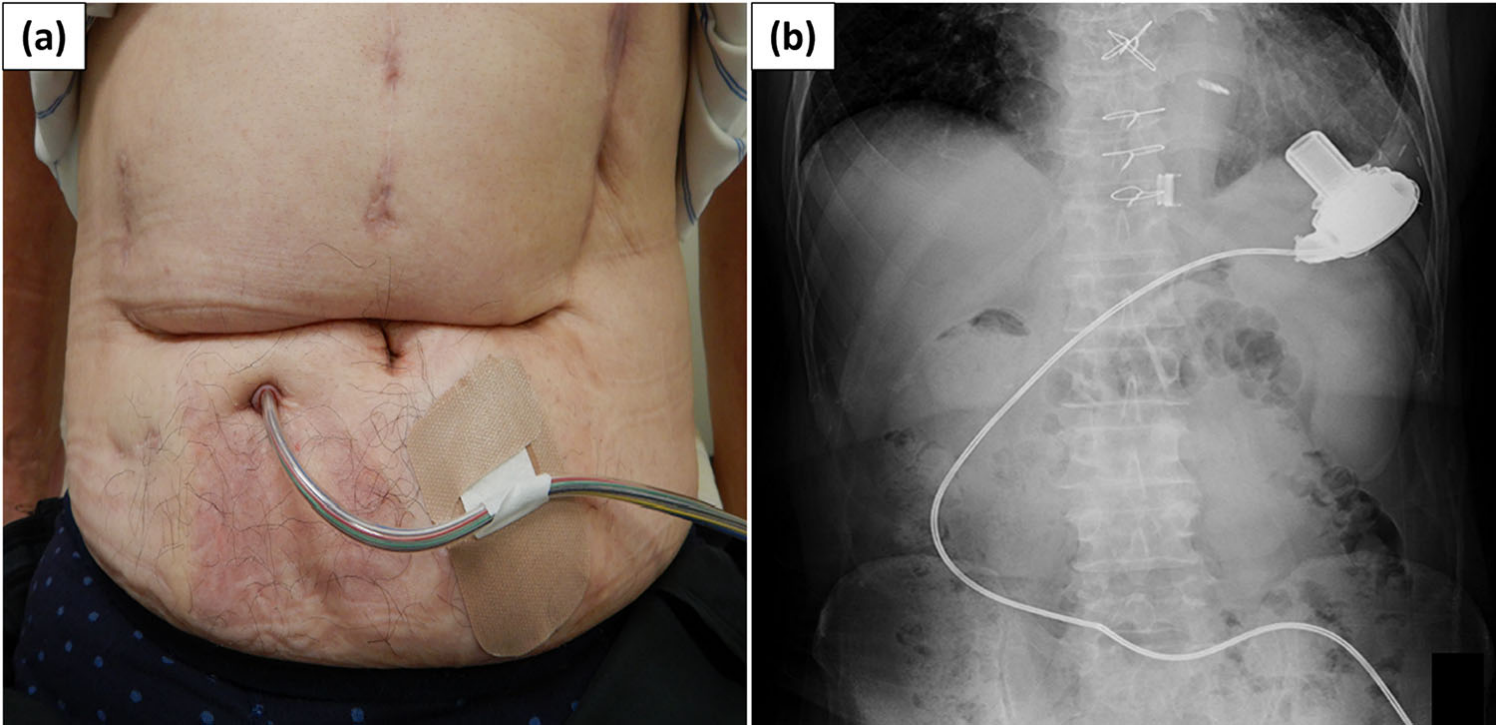
(a, b) The driveline is guided out of the body through the right hypochondrium, and radiographs show a HeartWare ventricular assist device positioned in the cardiac apex.

**FIGURE 2:**
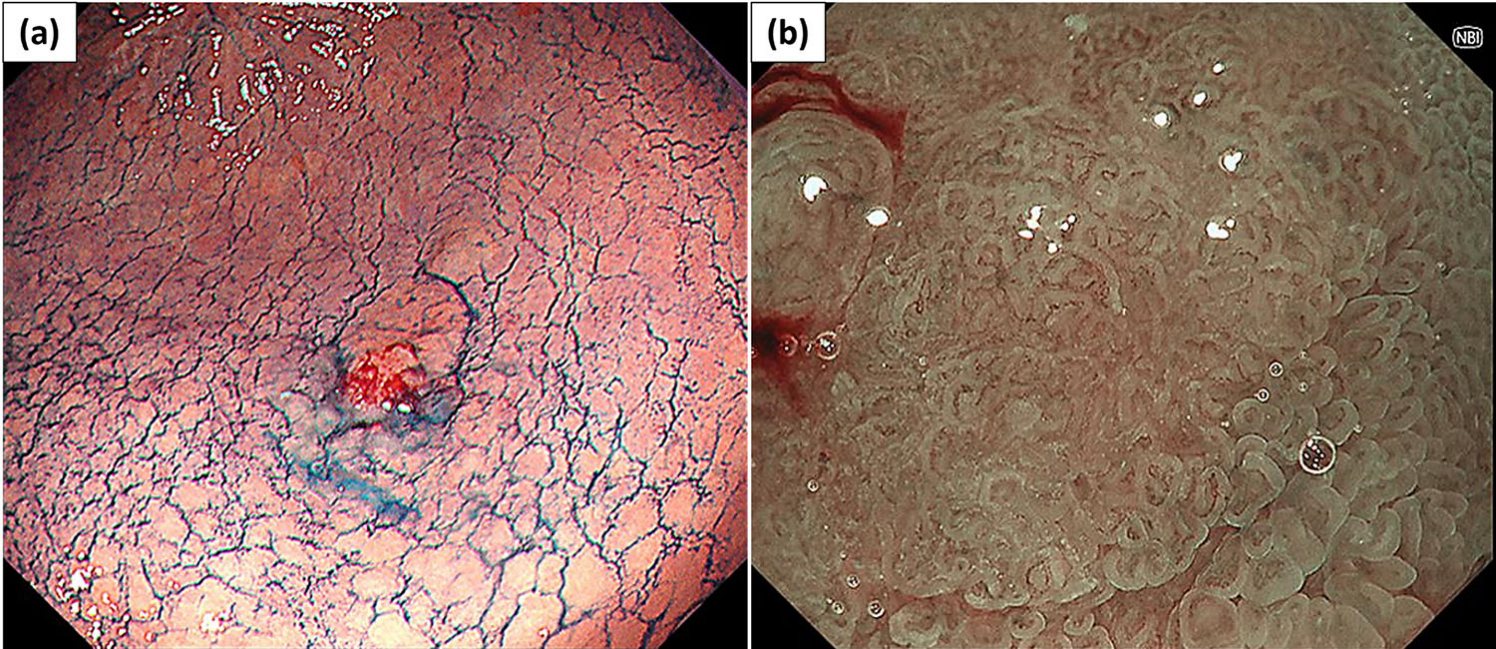
(a) Esophagogastroduodenoscopy revealed a 15‐mm flat‐elevated lesion at the greater curvature of the gastric angle under white light imaging with indigo carmine spraying. (b) The lesion was recognized as a demarcated brownish area with an irregular microsurface and microvascular patterns under narrow‐band imaging magnification.

A 69‐year‐old man who underwent ESD for early gastric cancer (EGC) 5 years prior visited our department for surveillance esophagogastroduodenoscopy. The patient had an LVAD (HeartWare ventricular assist device [HVAD]; Medtronic) implanted as DT for severe heart failure due to myocardial infarction 3 years prior (Figure 1). Esophagogastroduodenoscopy revealed a 15‐mm flat‐elevated lesion at the greater curvature of the gastric angle, and the biopsy revealed a well‐differentiated adenocarcinoma (Figure 2a,b).

The patient was scheduled for ESD. The prothrombin time (PT) activity at admission was 23%, the international normalized ratio (INR) was 2.41, and the activated partial thromboplastin time (APTT) was 42 s. Antithrombotic drugs (aspirin 100 mg, warfarin 3.5 mg) were discontinued and replaced with 10,000 units of heparin, following the advice of cardiovascular surgeons.

**FIGURE 3:**
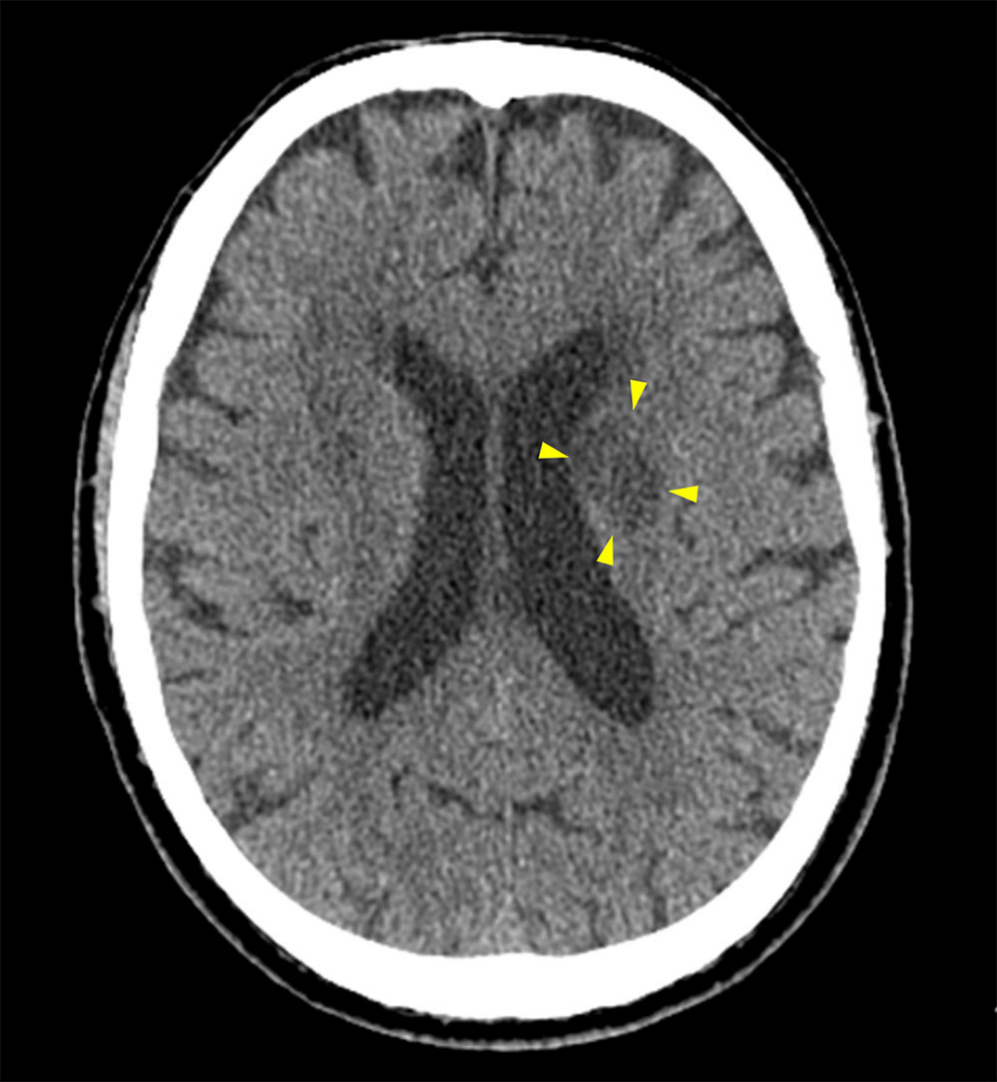
A computed tomography scan on day 5 revealed a low‐density area from the left corona radiata to the basal ganglia, and the patient was diagnosed with acute cerebral infarction.

On the second day, dysarthria and right upper‐extremity movement disorder appeared, and despite the change in medication, the PT‐INR was 2.01 and APTT was 45 s. Magnetic resonance imaging could not be performed because of the LVAD, and computed tomography on the day of onset showed only old infarcts in the left corona radiata, basal ganglia, and right posterior limb of the internal capsule; no new infarction site was noted. However, on the fifth day, computed tomography revealed a low‐density area extending from the left corona radiata to the basal ganglia (Figure 3), leading to a diagnosis of acute cerebral infarction.

Aspirin and warfarin were immediately restarted, and heparin infusion was discontinued after PT activity resumed. The ESD was postponed during the acute phase of the stroke. Thereafter, no worsening of the neurological abnormalities was noted, and a trend toward symptomatic improvement was observed. After consulting with cardiovascular surgeons and neurologists, the patient and his family members were again fully informed of the risks associated with ESD, and their consent was obtained.

**FIGURE 4:**
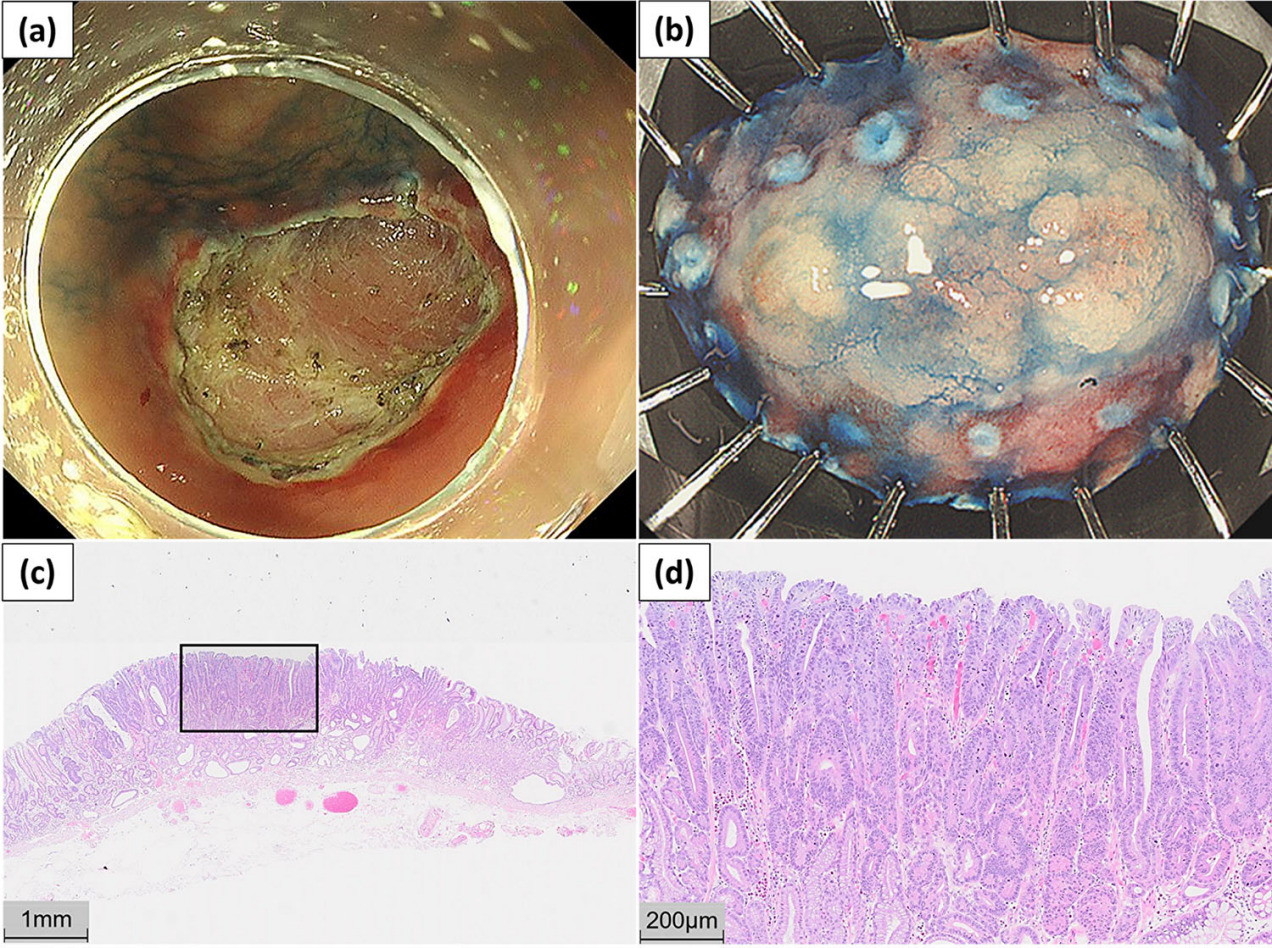
(a) Post–endoscopic submucosal dissection ulcer remaining after cauterization of the exposed vessels on the surface. (b) The endoscopic submucosal dissection specimen was a slightly elevated 14 × 9 mm lesion that was resected with sufficient margins. (c, d) The pathological diagnosis was a well‐differentiated adenocarcinoma, 0‐IIa, 14 × 9 mm, pT1a‐M, Ly0, V0, pUL0, pHM0, and pVM0.

Two months later, the ESD was performed under continuous warfarin administration (PT‐INR, 2.62; APTT, 45 s). Neurological manifestations were carefully monitored; however, no new abnormalities were observed. The warfarin was stopped only on the day of the ESD. During the procedure, the patient was carefully positioned so that the HVAD driveline was not dislodged or damaged, and a cardiovascular surgeon monitored the patient during the procedure for any abnormalities in the HVAD. Extracellular fluid was administered via intravenous infusion at a minimal dose to prevent heart failure. With the patient in the left lateral position an endoscope (GIF‐H290T; Olympus) was inserted under moderate intravenous sedation. After submucosal injection and an incision around the lesion, the lesion was dissected en bloc using a FlushKnife BT‐S (FUJIFILM) without intraoperative complications such as bleeding or perforation. After a successful resection, the exposed vessels on the ulcer surface were cauterized using a Coagrasper (Olympus; Figure 4a,b). Although no special devices or techniques were used for time reduction, the time for ESD from marking around the lesion to completion of the resection was 19 min, and the total operative time from endoscope insertion to removal was 27 min. No postoperative hematemesis or hemorrhage was observed.

The patient was started on a diet on post‐ESD day 2 and was discharged without any adverse events on post‐ESD day 6, following our hospital's clinical pathway. The pathological diagnosis was a mucosal well‐differentiated adenocarcinoma, and curative resection was considered achieved (Figure 4c,d).

## DISCUSSION

ESD is now widely performed as the standard curative treatment for EGC. A study examining the prognosis of 71 patients who remained untreated for >6 months after the diagnosis of EGC demonstrated a 63% rate of progression to advanced cancer at 5 years.^2^ Oh et al. also reported a median time of 34 months for untreated gastric cancer to progress from the T1 to T2 stage.^3^ These reports indicate the need for therapeutic intervention in patients with EGC with an expected long‐term prognosis.

In contrast, LVAD implantations for DT have recently increased, with Kirklin et al. reporting a 5‐year survival rate of approximately 40%.^1^ Considering the “era effect,” improved prognoses can be expected with advancements in devices and management. The two aforementioned reports regarding the nature of EGC did not specify whether the lesions were resectable by ESD; therefore, if limited to lesions eligible for ESD, the progression time to advanced gastric cancer may be longer. However, to consider ESD indications for patients with EGC, including those with LVAD, is necessary, because untreated gastric cancer progression can affect the quality of life and prognosis. Furthermore, we believe that ESD can be applied to patients with LVAD implanted not only as DT but as bridge‐to‐transplantation because EGC may conflict with the indication criteria for transplantation and require treatment. This first report of ESD is valuable in this context. Non‐cardiac surgery for patients with LVAD is reportedly feasible and safe^4^ despite the higher invasiveness of the procedure than ESD and the requirement for special care during the surgery. Contact or damage to the driveline and LVAD during surgery may lead to infection. As ESD does not directly contact them anatomically, the risk of infection is very low, except for intra‐abdominal infections caused by perforations. In the present case, the machine body limited the patient's ability to maintain a left‐lateral position during ESD, and the driveline required careful attention to avoid dislodging or damage.

In LVAD patients, stroke is among the most frequent complications, and anticoagulants are generally administered to mitigate the pro‐thrombotic tendency and thrombosis risk. In patients with an HVAD, the PT‐INR should be controlled at 2.0–3.0.^5^ Consequently, these patients are at high risk of gastrointestinal bleeding^6^, ^7^ due to the use of antithrombotic agents.

The Japanese gastrointestinal endoscopy guidelines for patients on antithrombotic therapy recommend continuing aspirin and switching warfarin with heparin when gastric ESD is performed in patients on dual antiplatelet and anticoagulant therapy.^8^ However, these guidelines do not assume ESD in patients with LVAD. Cho et al. reported that 57 (12%) of 477 patients with LVAD had cerebral infarctions. Of the 43 patients who received antithrombotic therapy, 15 (35%) did not experience a sufficient antithrombotic effect, which may have been the cause of their stroke.^9^ Simultaneously, Yalini et al. reported no cases of perioperative thrombotic events or LVAD thrombosis among 17 LVAD patients who underwent standby laparoscopic surgery when warfarin was discontinued 3–4 days before surgery and preoperatively replaced with intravenous heparin.^4^

In the present case, warfarin was replaced with heparin; however, the patient developed cerebral infarction. Although the therapeutic APTT was unachieved, the PT‐INR was within the range of 2.0–3.0, suggesting that warfarin's antithrombotic effect was still present, and whether the subtherapeutic anticoagulation effect alone was responsible for the stroke remains unclear. Therefore, determining the optimal perioperative management for patients undergoing LVAD implantation is difficult. However, strict monitoring of antithrombotic parameters is required to avoid cerebral infarction. Considering thromboembolism and bleeding risk associated with heparin replacement during the transition period, ESD with continuous warfarin administration may be appropriate as long as PT‐INR can be controlled within the optimal range.

In summary, this case report provides useful information about the feasibility and perioperative management of endoscopic intervention for patients with LVAD.

## CONFLICT OF INTEREST STATEMENT

None.
